# Advancements in culture technology of adipose-derived stromal/stem cells: implications for diabetes and its complications

**DOI:** 10.3389/fendo.2024.1343255

**Published:** 2024-04-12

**Authors:** Yinze Shi, Xueyang Yang, Jie Min, Wen Kong, Xiang Hu, Jiaoyue Zhang, Lulu Chen

**Affiliations:** ^1^ Department of Endocrinology, Union Hospital, Tongji Medical College, Huazhong University of Science and Technology, Wuhan, China; ^2^ Hubei Provincial Clinical Research Center for Diabetes and Metabolic Disorders, Wuhan, China

**Keywords:** adipose-derived stem/stromal cells, monolayer culture, three-dimensional culture, diabetes mellitus, diabetic foot ulcer

## Abstract

Stem cell-based therapies exhibit considerable promise in the treatment of diabetes and its complications. Extensive research has been dedicated to elucidate the characteristics and potential applications of adipose-derived stromal/stem cells (ASCs). Three-dimensional (3D) culture, characterized by rapid advancements, holds promise for efficacious treatment of diabetes and its complications. Notably, 3D cultured ASCs manifest enhanced cellular properties and functions compared to traditional monolayer-culture. In this review, the factors influencing the biological functions of ASCs during culture are summarized. Additionally, the effects of 3D cultured techniques on cellular properties compared to two-dimensional culture is described. Furthermore, the therapeutic potential of 3D cultured ASCs in diabetes and its complications are discussed to provide insights for future research.

## 1 Introduction

Stem cell-based therapy, including pluripotent stem cells (PSCs) and mesenchymal stromal/stem cells (MSCs), represents an innovative therapeutic strategy that capitalizes on the distinctive characteristics of stem cells, such as self-renewal and differentiation capabilities, to facilitate the regeneration of impaired cells and tissues within the body or the substitution of these cells with new, healthy, and fully functional cells by delivering exogenous cells (1).

PSCs are characterized as a type of self-renewing cells capable of differentiating into diverse cellular phenotypes originating from the three germ layers of the body (2). PSCs, including embryonic stem cells (ESCs) and induced pluripotent stem cells (iPSCs), has revolutionized stem cell research and cell-based therapy (3). Nonetheless, the utilization of ESCs is constrained by ethical considerations, the possibility of immunological rejection, and the potential for tumorigenicity (1, 4). In contrast, iPSC technology overcomes ethical dilemmas associated with ESCs derived from human embryos, enabling the creation of patient-specific pluripotent stem cells. However, iPSCs are generated through the ectopic expression of pluripotency factors, often facilitated by viral vectors or non-viral reprogramming factors, which may lead to genomic instability (5, 6). Besides, iPSCs have been shown to elicit T cell-dependent immune response (7) and promote tumor formation (3, 8). Consequently, thorough safety assessments are imperative prior to iPSC transplantation.

Mesenchymal stromal/stem cells (MSCs) are adult stem cells with multipotent capabilities, including self-renewal (albeit limited in vitro) and differentiation into various mesenchymal lineages (9, 10). MSCs have been shown to overcome ethical concerns and mitigate the risk of mutational side effects associated with. Additionally, MSCs exhibit the lowest immunogenicity compared to other stem cell types, making them a favorable option for clinical use (11). In the field of organ and cell transplantation, MSCs have been utilized for their secretion of growth factors and immunoprotective cytokines. Their ability to differentiate into various cell types has been harnessed for applications in tissue engineering (12). Among these, adipose-derived MSCs (ASCs) are particularly advantageous due to their larger storage with less discomfort and damage to the donor site, easier accessibility without significant donor site morbidity, higher proliferation ability, fewer ethical concerns, and fewer immunological rejection (11, 13, 14). Furthermore, some growth factors and immunomodulators are more actively secreted in ASCs (13). Therefore, ASCs may be a better candidate for clinical application in theory.

Diabetes mellitus (DM) is a severe and chronic disease characterized by elevated blood glucose levels resulting from aberrant islet β-cell biology and insulin action (15). In 2021, the global population living with diabetes reached 529 million (15). Given β-cell dysfunction across various types of DM, most patients ultimately require insulin therapy (16–18). However, this therapy is frequently limited by individual factors, such as weight gain, fear of needles and lifestyle considerations, all of which contribute to poor glycemic control. Furthermore, insulin therapy cannot reverse β-cell damage and progress of diabetes, or replicate the normal physiological state. In recent clinical applications, pancreatic islet and cell transplantation have emerged as potential strategies (19). However, these procedures have numerous challenges, including the scarcity of suitable donors, surgical complexities, side effects associated with immunosuppressive agents as well as exhaustion of transplanted organs and cells (11). Furthermore, it is necessary to maintain β-cell function and blood glucose homeostasis, otherwise life-threatening complications are likely to occur (20).

In the treatment of diabetes and its complications, ASCs have been used due to their inherent attributes such as self-renewal capacity, differentiation potential, homing mechanism and immunosuppressive property (11, 21). Furthermore, three-dimensional (3D) cultured cells are studied to prolong the lifespan of transplanted cells and enhance their pro-healing functions in unfavorable environments (22–25). Recent literature provides numerous strategies for obtaining 3D cultured ASCs (26). These cells possess enhanced abilities to maintain their stemness and display multilineage plasticity compared to cells cultured in adhesion (26). Moreover, 3D cells more closely mirror biological processes compared to cells cultured in traditional monolayers, driving the need for the development of 3D culture, including spheroids, organoids, organ-on-a-chip models, and bioprinting (27–29).

Despite being an emerging and rapidly developing technology, there is currently no standardized method for ASC culture and no summary for the research of 3D cultured ASCs in diabetes and its complications. In this review, we summarize current knowledge about monolayer ASC culture techniques, with a particular emphasis on the influential factors during culture. Additionally, the effects for cellular properties of 3D cultured methods compared to two-dimensional (2D) culture is described. Furthermore, the therapeutic potential of 3D cultured ASCs in diabetes and its complications are discussed to provide insights for future research.

## 2 Nomenclature of adipose-derived stromal/stem cells

There is inconsistency in the nomenclature of this plastic adherent cell population isolated from adipose tissue (30). In 2006, the International Society for Cellular Therapy (ISCT) acknowledged the “inconsistencies and ambiguities” of the term “mesenchymal stem cells” and recommended a new designation: multipotent mesenchymal stromal cells (31). It is recommended to use the abbreviation “MSCs” in conjunction with extra information like AD-MSCs (9) (adipose tissue-derived MSCs) or MSC(AT) (32) and clearly define stem cells or stromal cells in terms of their function (9). Additionally, Caplan proposed the term “medicinal signaling cells” due to their therapeutic actions, which include homing to the site of injury and secreting regenerative and immunomodulatory factors (33). Despite the advocacy for standardization in nomenclature, it is still most common to refer to MSCs as “mesenchymal stem cells”, followed by “mesenchymal stromal cells” or a combined use of “stem/stromal” terms (34). In this review, following search terms for this kind of cells were adopted: “adipose-derived stromal cells”, “adipose-derived stem cells”, “adipose-derived stromal/stem cells”, “ASCs” and “ADSCs”, and having no limitation to the human or animal species.

## 3 Monolayer culture techniques

**Figure 1:**
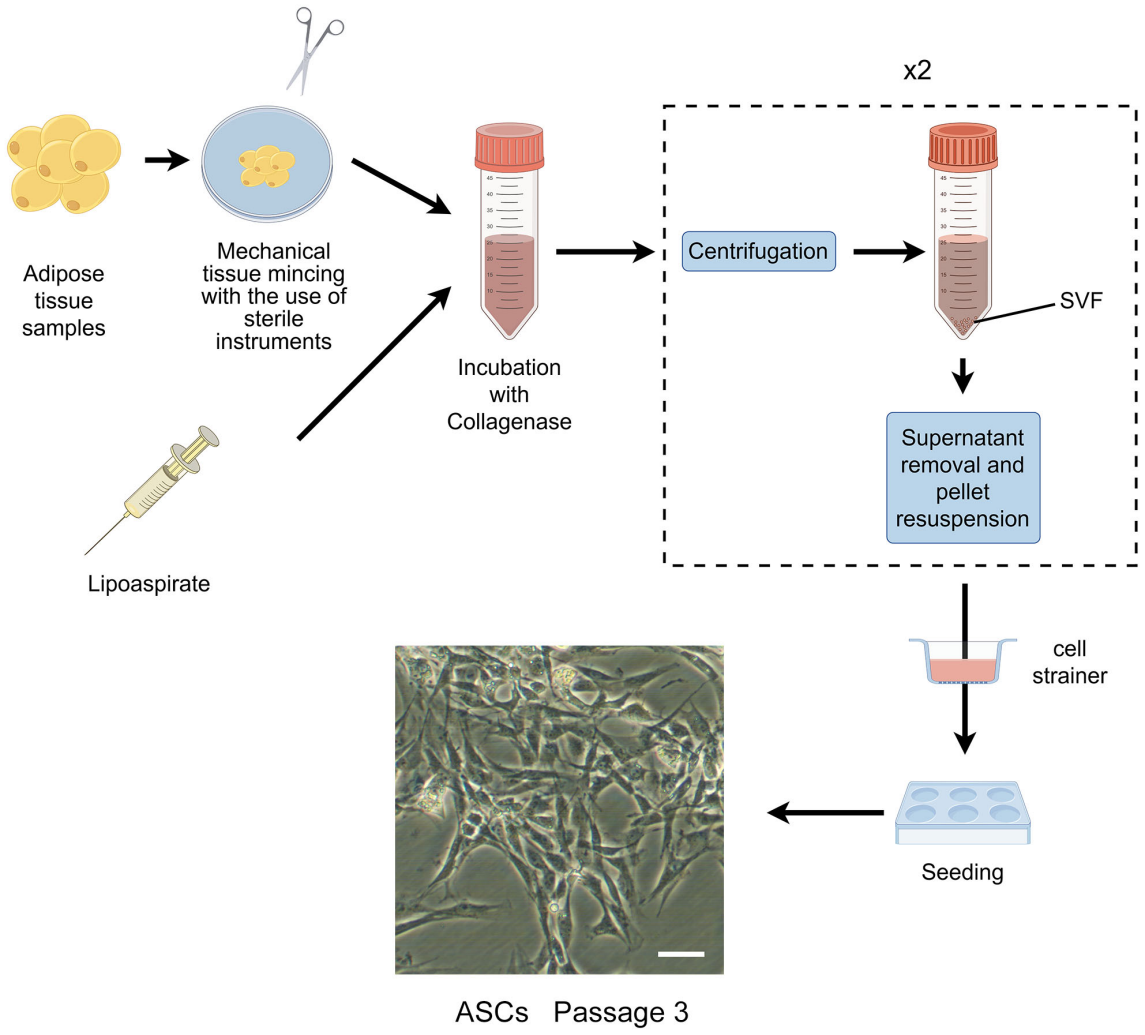
The isolation process of ASCs. The cells showed are isolated from rat’s inguinal adipose tissue. Scale bar, 200μm. SVF, stromal vascular fraction; ASCs, adipose-derived stromal/stem cells.

In the 1960s, Rodbell and Jones pioneered the initial method of isolating cells from adipose tissue (35–37). The researchers isolated stromal vascular fraction (SVF) from rat fat pads, which contained heterogeneous cells. In the final step, adherent plastic cells within the SVF were selected and enriched for “preadipocytes”. In 2001, ZUK et al. obtained a fibroblast-like cell population or a processed lipoaspirate from human lipoaspirate. They determined these cells could differentiate into adipogenic, osteogenic, chondrogenic, and myogenic cells in vitro, which opened up new avenues for MSC research (38). The isolation and culture process of ASCs is shown in **Figure 1**.

**Figure 2:**
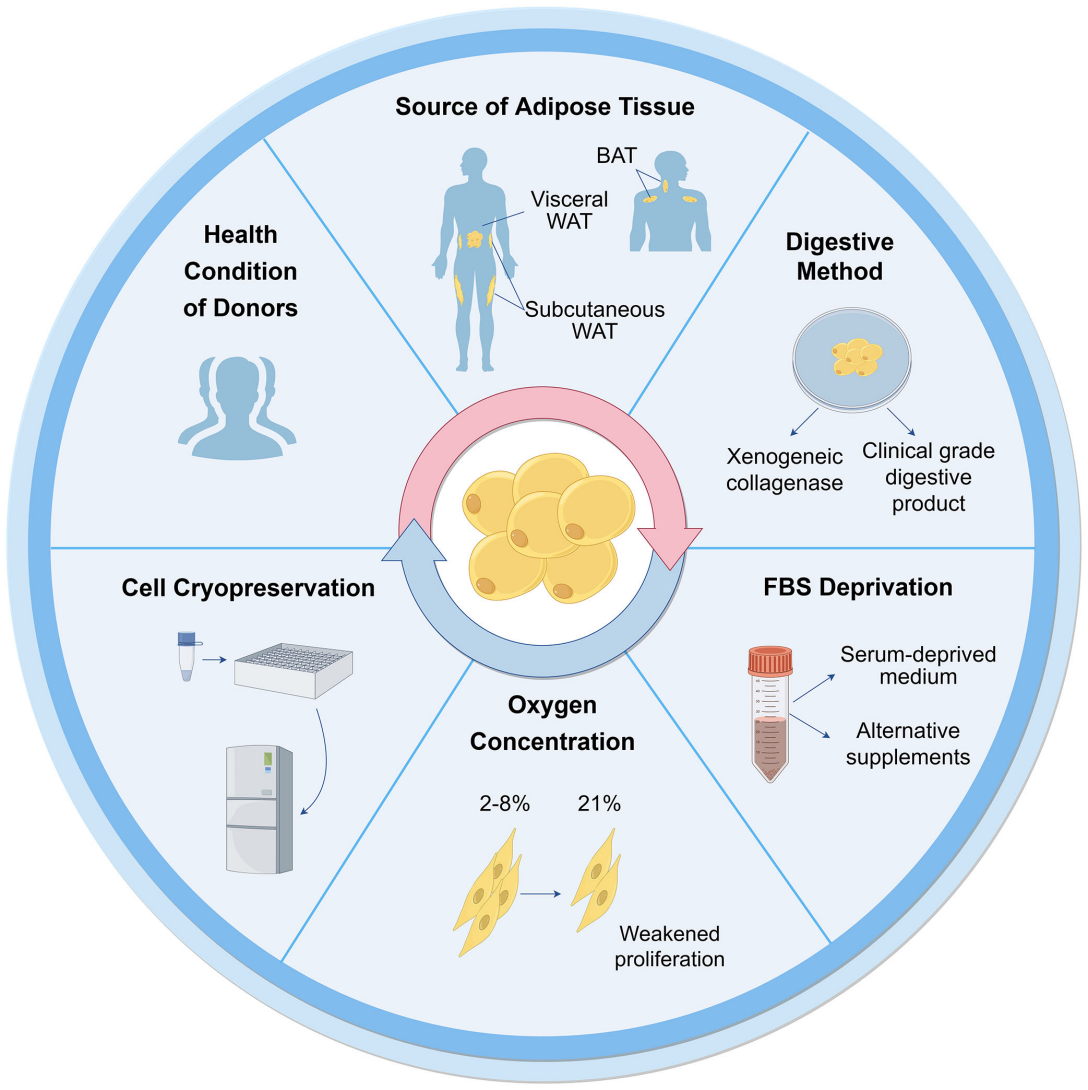
Influential factors on biological functions of ASCs during culture. Many aspects are reported to influence ASC culture and their biological functions. These can broadly be divided into the sources of tissues and cells, techniques of isolation, culture and cryopreservation. WAT, white adipose tissue; BAT, brown adipose tissue; FBS, fetal bovine serum.

The characterization of ASCs involves fulfilling specific criteria related to cellular morphology (39, 40), immune-phenotypic (10), and differentiation capacity (10, 31). As high quality of cells is the prerequisite for their application, various factors that may influence their biological functions during culture have been proposed (**Figure 2**).

### 3.1 Tissue and cell sources

#### 3.1.1 Health conditions of donors

Cells can be obtained from healthy donors or individuals with varying degrees of diabetes, obesity, and other chronic diseases. The use of autologous and allogeneic ASCs should be carefully considered. Autologous cells have advantages in terms of histocompatibility and infectious concerns (41), but their functionality may be compromised in an unhealthy environment. ASCs derived from diabetic donors have shown reduced proliferation ability and paracrine activity compared to autologous ASCs from healthy individuals (42–44), but they still hold potential in cell therapies (45–47). Additionally, Obesity has an adverse impact on ASCs, resulting in defective functionalities and properties (48). ASCs from individuals with obesity exhibit decreased telomerase activity and telomere length (49). There are no significant differences observed in ASCs between oncological patients and healthy subjects (50, 51). However, ASCs from donors exposed to radiotherapy and chemotherapy exhibit altered cell migration, proliferation, and differentiation capacity (48). The outcomes are also correlated with other demographics, such as age, gender, and ethnicity (52).

#### 3.1.2 Types of adipose tissue

White adipose tissue (WAT) mainly exists in two types: subcutaneous and visceral adipose tissue. ASCs obtained from subcutaneous (S-ASCs) and visceral adipose tissue (V-ASCs) share similar cell viability and surface markers but differ in motility, secretory function, and expression of stemness-related genes (53). However, S-ASCs have a greater differentiation capacity to adipogenic and osteogenic cells, and V-ASCs proliferate slower, require stronger stimulation for differentiation (54), and secrete higher levels of inflammatory cytokine such as interleukin (IL)-6, IL-8 and tumor necrosis factor (TNF)-α (55). Wada et al. (56). also found that V-ASCs and S-ASCs release inflammatory and angiogenesis cytokines differently. Moreover, ASCs in the superficial layer, located closer to the dermis, exhibited hyperplastic and angiogenic capacities, while ASCs in the deep layer were characterized by inflammatory properties similar to V-ASCs (27).

Furthermore, studies have shown the presence of ASCs derived from brown adipose tissue (BAT) (57, 58). The characteristics of ASCs derived from BAT differ from those of WAT, particularly, the expression of myogenic factor 5 (Myf5) and myogenic origin. In these cells, gene expression profiles are unique, particularly the higher expression of genes associated with BAT including uncoupling protein-1 (UCP1), peroxisome proliferator-activated receptor gamma coactivator 1-alpha (PGC-1α), PR domain containing 16 (PRDM16), and CAMP responsive element binding protein one (CREB1). Therefore, the tissue and cell sources should be considered for further application.

### 3.2 Isolation and culture

#### 3.2.1 Collagenase digestion

The first crucial step in obtaining cells from adipose tissue is cell isolation. Currently collagenase digestion remains the most common method to obtain cells due to its simplicity and high cell purity (41, 48). However, the use of xenogeneic collagenase may lead to pathogen transmission and immune response in vivo. To be considered safe, the development of clinical grade digestive products is crucial for the isolation of ASCs. Carvalho et al. demonstrated that several alternative enzyme products, including Collagenase NB 4 Standard Grade (NB4) [Serva], Collagenase Type 1 (CLS1) [Worthington], Collagenase (Animal Origin Free)-A (CLSAFA) [Worthington], and Liberase [Roche], were equally effective as research-grade products (59). Kølle et al. implemented a clinical trial using cells which were isolated by clinical collagenase NB 6 (60).

#### 3.2.2 Serum deprivation

Fetal bovine serum (FBS) is another important consideration for ASC culture and application, similar to xenogeneic collagenases. The available studies showed that human ASCs (hASCs) maintain their stemness in serum-deprived medium (61). In the absence of FBS for 48 hours, hASCs showed reduced metabolism and proliferation, but maintained the expression of crucial surface markers, without undergoing apoptosis or necrosis (51). Human ASCs cultured in STK2 (a chemically-defined serum-free medium) exhibited enhanced proliferation, elevated expression of MSC surface markers, and diminished cell aging compared to those cultivated in media supplemented with FBS (62). According to these observations, FBS deprivation does not cause impacts that would prevent cellular clinical application.

Other alternative supplements have been investigated as potential substitutes for FBS. Human platelet lysates (HPLs) could serve as a superior supplement. They were found to augment the proliferative capacity of hASCs in comparison to FBS, while simultaneously preserving their untransformed state and differentiation ability (63, 64). Kocaoemer et al. observed that hASCs cultured in medium supplemented with either thrombin-activated platelet rich plasma (tPRP) or pooled human serum (HS) exhibited similar properties, although a reduction in adhesion was observed in cells cultured in tPRP-supplemented medium (65). According to the whole genome gene analysis, 90 genes were significantly expressed more in hASCs cultured in FBS-supplemented medium (66).

#### 3.2.3 Oxygen concentration

As the oxygen concentration of adipose tissue in vivo is 2%-8%, ASCs exist in a relatively low-oxygen microenvironment (67, 68). However, most ASCs are cultured under normal oxygen conditions (21% oxygen concentration) in vitro. Human subcutaneous ASCs cultured in hypoxic conditions in vitro exhibited increased proliferation rates and secretion of growth factors (69). Tirza et al. discovered weakened proliferation ability, increased accumulation of reactive oxygen species (ROS), and genetic instability of rat visceral ASCs cultured under normal oxygen experienced, which could be improved by lowering the culture temperature (67).

#### 3.2.4 Cell cryopreservation

Despite the diminished cell viability and lower colony-forming-unit percentages observed in cells derived from cryopreserved lipoaspirate compared to fresh lipoaspirate-derived cells, the viable cells that remained exhibited preserved adhesive and proliferative properties (70), which could counteract the negative effect with continued cell growth (71). After prolonged cryopreservation at 70°C, the number of viable cells decreased as well as their viability (71). A cryopreservation medium containing HS, HS albumin, or knockout serum replacement did not affect the gene expression, differentiation ability, and immunophenotype of hASCs for a duration of 3-4 freeze-thaw cycles, but significantly reduced the proliferation. Thus, it has been recommended that cells for clinical application should not undergo more than two freeze-thaw cycles (72).

In summary, isolation and culture methods can affect ASCs properties, therefore, there is still a need to look for appropriate culture protocol that will provide the right number and characteristics of ASCs without affecting their therapeutic potential for clinical application.

### 3.3 Cellular senescence and potential interventions

Cellular senescence, also called aging, has always been an obstacle to the development of MSC therapy. Some studies confirmed the stability of ASCs during a certain period (usually up to the sixth or seventh passage) (51, 73, 74). However, Yin et al. found that hASCs rapidly underwent replicative senescence and lost stem cell properties over 21 days by current 2D culture (75). During long-term culture, senescent cells experience a cessation in proliferation, and exhibit distinct morphological and physiological features, including enlarged nuclear and cytoplasmic volumes, heightened β-galactosidase enzyme activity, decreased expression of β cell-specific Moloney murine leukemia virus integration site 1 (Bmi-1), telomere shortening and accumulation of ROS (76, 77). The stability and safety of ASCs should be considered in application, thus, many research efforts have been enhanced to address the problem of cellular aging.

Immortalization techniques have been shown to overcome senescence in primary cells (78). Kang et al. discovered that ectopic expression of telomerase reverse transcriptase (TERT) in non-human primates ASCs enabled cells to maintain proliferative potential and multipotent differentiation ability (79). Tchkonia et al. generated preadipocyte strains from single abdominal subcutaneous, mesenteric and omental human preadipocytes through stable expression of human TERT (hTERT). These strains were capable of repeated subculturing and maintained the capacity for differentiation, as well as the specific dynamic characteristics of fat depot cells (80). Wolbank et al. found hTERT overexpression generated ASC lines (ASCs^hTERT^) exhibited continuous growth and showed minimal changes in morphology, surface marker profile, karyotype, immunosuppressive capacity and differentiation potential (81). Similarly, Shamsi and Tseng developed protocols for immortalizing brown and white preadipocytes (82). Furthermore, researchers have cultured ASCs with TERT expression to conduct further researches in regenerative medicine and other medical fields (79, 83–85).

Furthermore, Tátrai et al. found that human ASCs^hTERT^ and ASCs generated by the co-transduction of hTERT and Bmi-1 retained MSC features and did not senesce, whereas ASCs generated by the overexpression of Bmi-1 exhibited limited replicative potential. Notably, a subpopulation of ASCs^hTERT^ also acquired aberrant karyotype and showed signs of transformation after long-term culture (86).

However, Balducci et al. found that hTERT alone failed to immortalize hASCs. Moreover, hASCs that were co-transduced with hTERT and human papillomavirus (HPV)-E6/E7 were successfully immortalized and could secrete significant amount of hepatocyte growth factor (HGF) and vascular endothelial growth factor (VEGF), albeit with reduced differentiation properties and some chromosomal aberrations (87). Darimont et al. demonstrated that co-transduction of hTERT and HPV-E7 enabled human preadipocytes to extend their lifespan and maintain their capacity for differentiation (88).

The overexpression of simian virus 40 large T antigen (SV40T) has been widely employed as a strategy to overcome replicative senescence in human primary cells. However, it was found that the adipogenic differentiation process was blocked by SV40T expression in 3T3-F442A cells (89). Human ASC lines operated by co-transduction of hTERT and SV40T underwent chromosome aberration, deviated from the normal MSC phenotype, and lose the ability of differentiate (86, 87).

Although there were variations in results across different studies, it is generally established that cell immortalization can be achieved through gene editing technology (**Table 1**). Notably, the possibility of karyotype variation should be taken into consideration in these immortalized cells constructed by gene editing technology.

**Table 1:** Immortalization of ASCs through gene editing technology.

Transduction	Proliferative potential	Multipotent differentiation	Secretion ability	Karyotype
TERT	√	√	√	Slightly changed
Bmi-1	Having decelerated growth and completely arrest in later-passage	√	√	Normal
TERT and Bmi-1	√	√	√	Normal
TERT and HPV-E6/E7	√	Slightly reduced	√	Having some chromosomal aberrations
TERT and SV40T	√	Blocked	Unknown	Having multiple chromosome aberrations

TERT, telomerase reverse transcriptase; Bmi-1, B cell-specific Moloney murine leukemia virus integration site 1; HPV, human papillomavirus; SV40T, simian virus 40 large T antigen.

“√” represents maintenance or enhancement.

## 4 3D culture techniques

Significantly, advancements in stem cell and 3D culture technologies have enabled the creation of cellular models that accurately mimic the histological, molecular, and physiological characteristics of tissues and organs (29). The formation of 3D cultures relies on the self-organization and differentiation of cells, as well as signaling cues from the extracellular matrix (ECM) and conditioned media (90).

### 4.1 Cell types

3D cultures are typically self-assembled in vitro 3D structures derived from primary tissues or various types of stem cells, including MSCs, iPSCs, and ESCs. Various cell types exhibit distinct developmental pathways, underscoring the importance of selecting an appropriate initial cell population for the successful establishment of organoid cultures. 3D culture of ESCs has not been a priority due to their ethical concerns. 3D culture models derived from MSCs have been shown to highly recapitulate the homeostasis and regenerative capacity of the tissue of origin (91). Conversely, models derived from iPSCs often hardly recapitulate the adult tissue stage, instead resembling the fetal tissue stage (92, 93). 3D culture models derived from ASCs are generated without genetic modification by transcription factors, unlike those derived from iPSCs (94). Moreover, ASC exhibit immune privileged properties, and accordingly show excellent safety for allogeneic transplantation in multiple human clinical trials (4). Therefore, ASCs is a cell type with great potential and advantages in 3D culture technology.

### 4.2 Effects of culture techniques on cellular properties

Despite numerous studies, there is no standardized method for 3D ASC culture. It is necessary to comprehend the impact of different 3D culture techniques on cellular properties in contrast to traditional 2D culture.

#### 4.2.1 Cell viability and stemness

The stemness properties of MSCs are retained in the in vivo microenvironment, which includes soluble growth factors, cell-cell interactions and cell-matrix interactions (95, 96). Increasing evidence has indicated that the cellular microenvironment significantly influences stemness properties (95, 97). In comparison to conventional monolayer cultures, 3D cultured methods provide a cellular niche that more closely resembles the in vivo microenvironment (98).

Existing techniques for ASC culture can be categorized into scaffold-free and scaffold systems (26, 99). The conventional scaffold-free culture techniques, such as the use of low adhesion plates, hanging drops, and spinner flasks, have been shown to impact the viability and stemness of ASCs.

Low adhesion plate culture method involves the formation of spheroids by suspending cells on a surface with low adhesion properties. Guo et al. successfully generated 3D spheroids using non-adhesive agarose Petri dishes. This method was found to overcome poor post thaw cells and improve the viability and neural differentiation potential of hASCs (100). Similarly, Coyle et al. conducted a study examining hASC spheroids with various sizes and demonstrated the enhanced viability of spheroids was achieved through anaerobic glycolysis in conditions of increased glucose availability and decreased oxygen levels (101). Di Stefano et al. conducted a comparative analysis of hASCs cultured in ultralow culture flasks and hASCs with 2D primary cultures. Their study identified distinct molecular expression patterns of genes associated with stemness, as well as genes related to anti-aging, oxidative stress, and telomeres maintenance of hASCs (102). Rybkowska et al. conducted a study in which 3D hASC spheroids were cultured using antiadhesive plates. However, they observed that the spheroids exhibited slightly lower viability, reduced proliferation rates, but higher expression of stemness-related transcriptional factors compared to cells cultured in monolayer. Additionally, the 3D culture resulted in increased mitochondrial DNA content, oxygen consumption rate, and extracellular acidification rate. Elevated levels of ROS and decreased intracellular lactic acid levels were also detected (103).

The hanging drop method technique capitalizes on the intrinsic tendency of cells to self-assemble into three-dimensional aggregates needless of scaffolding. A drop is formed within an inverted plate and held in place due to surface tension. Jin et al. utilized the hanging-drop technique to produce hASC microtissues in a smooth muscle inductive medium supplemented with human transforming growth factor β1, and subsequently bioprinted these induced microtissues onto a 3D framework. The microtissues retained their phenotypic characteristics post-bioprinting. Cell viability and proliferation within the 3D microtissues were consistently superior in comparison to the traditional single-cell bioprinting approach (104).

The spinner flask facilitates the generation of fluid flow, which discourages cellular adhesion and facilitates cellular aggregation. Bangh et al. placed hASC spheroids in spinner flasks under 1% oxygen. The spheroids exhibited faster growth rates compared to monolayer cultures. Additionally, they observed an upregulation of survival factors in response to the spheroid size (105).

Another commonly employed approach involves seeding stem cells into scaffolds that mimic the ECM of native tissues, which can be fabricated using biologically derived or synthetic materials. Natural scaffolds consist predominantly of collagen, fibrin, gelatin, vitronectin, laminin, alginate, hyaluronic acid (HA), or decellularized materials, while synthetic scaffolds may consist of materials such as polyesters, polyethers, polyethylene glycol, and polylactic acid (PLLA) (106). Several studies have investigated the use of different hydrogels to create ASC spheroids, utilizing commonly used materials in tissue engineering such as HA and chitosan, resulting in enhanced stemness gene expression compared to traditional adhesion plate cultures (107–110). A poly(ethylene glycol) (PEG) hydrogel microwell pattern was fabricated on a poly(N-isopropylacrylamide) hydrogel substrate to regulate the size of spheroids. The viability of hASC spheroids exceeded 97.5% (111).

Based on mechanical structure or new systems, various novel techniques were devised to create 3D ASC structures with enhanced viability, increased stemness, and enhanced differentiation capabilities, such as the following: A switchable water-adhesive, super-hydrophobic nanowire surface (112); microgravity bioreactors (113); microwell plates employed with gelatin microparticles (114); microfabricated porous tissue strands (pTSs) (115); a method defined “all-in-one platform” with hydrogels with an embossed surface (HES) (116); gelatin hydrogels with microbial transglutaminase (mTG) (117); and TeSR-E8 medium (a highly chemically defined medium) in conventional tissue culture polystyrene dishes (118). Furthermore, Labriola et al. utilized polymer-based, cell mimicking microparticles (CMMPs) to deliver distinct, stable mechanical cues to hASCs in 3D spheroid culture. Mechanically tuned CMMPs controlled whole-spheroid mechanical phenotype and stability but minimally affected differentiation response (119).

Based on the findings of these studies, it is evident that the majority of research indicates that 3D culture enhances cell viability, stemness, proliferation rate, and metabolic functions, with only a few exceptions showing a decrease in cell viability.

#### 4.2.2 Differentiation ability

Multilineage differentiation potential of ASCs towards both mesenchymal and non-mesenchymal lineage cells have been reported, particularly towards adipogenic, chondrogenic, and osteogenic lineages, which can be facilitated by the introduction of lineage-specific factors (120).

Adipogenic differentiation: Decellularized adipose tissue (DAT) based hydrogels have been demonstrated to closely replicate the native ECM environment, effectively inducing adipogenic differentiation and promoting the proliferation of hASCs (121, 122). In a study by Zhang et al., hASC spheroids cultured in a microgravity bioreactor exhibited enhanced stemness properties and adipogenic differentiation potential compared to monolayer culture (113). Hoefner et al. cultured hASC spheroids in growth cell media under agitation at 50 revolutions per minute. After a brief 2-day induction period for adipogenic lineages, it was observed that ASC spheroids exhibited enhanced differentiation capacity within their own ECM when compared to traditional 2D cultures (123). These findings suggest that utilizing 3D ASC culture may be a promising approach for adipose tissue engineering applications.

However, Rumiński et al. reported that hASC spheroids seeded in 96-well sterile round-bottom culture plates and subjected to gentle rotation on a rotary shaker displayed reduced adipocyte differentiation (124). The elastin-like polypeptide (ELP)-polyethyleneimine (PEI) coated surface was demonstrated a suitable cell culture material (125). However, the study conducted by Turner et al. revealed that triglyceride accumulation was less pronounced in hASC spheroids seeded on ELP-PEI coated surfaces compared to 3T3-L1 adipocytes, correlated with smaller average spheroids, suggesting a relatively slower differentiation process (126).

Chondrogenic differentiation: Yoon et al. employed the spinner flask method to illustrate that 3D hASC spheroids exhibit enhanced chondrogenic capabilities when cultured in a specific differentiation medium as opposed to monolayer culture (127). Tsai et al. employed mTG, an enzyme with high specificity across a broad temperature range, to crosslink gelatin. The evaluation of differentiation potential revealed that hASC spheroids within the 3D gelatin/mTG hydrogel demonstrated heightened activity, particularly in adipogenesis and chondrogenesis, in comparison to the cell suspension group (117). Furthermore, when comparing hASC spheroids cultured using microwell techniques to ASCs cultured in a 2D monolayer, it was observed that cell survival and chondrogenic potential were enhanced, while apoptosis was diminished. Injecting hASC spheroids exerted enhanced regenerative capabilities for articular cartilage and effectively halted the advancement of surgically induced osteoarthritis through the paracrine mechanism of action, when compared to ASCs in single-cell suspension (128).

Osteogenic differentiation: Gurumurthy et al. illustrated that 3D hASCs cultivated on ELP-PEI scaffolds exhibited a heightened propensity for differentiation towards the osteogenic lineage in comparison to 2D cultures (129). Human ASCs were cultured in 3D systems devoid of bioactive material components: spheroids and polystyrene scaffolds. Alkaline phosphatase activity, a marker of early osteogenesis, exhibited increased levels in ASC spheroids and ASC-seeded scaffolds in comparison to 2D cultures. The expression of the osteoblast marker, including Runt-related transcription factor 2, and osterix and integrin binding sialoprotein was significantly up-regulated in spheroids compared to polystyrene scaffolds and 2D culture (124). Kim et al. conducted a study to evaluate the osteogenic potential of hASCs in 2D and 3D culture environments. Through comprehensive analysis of transcriptome sequencing data, they identified an upregulation of genes associated with skeletal development, bone formation, and bone remodeling processes in hASCs cultured in concave microwells (130).

Differentiation into other lineages: Cheng et al. utilized chitosan films to form hASC spheroids, which, when cultured in appropriate induction media, exhibited enhanced differentiation capabilities, including differentiation into neuron and hepatocyte-like cells (131). Guo et al. observed an increased capacity for neural differentiation in 3D hASC spheroids cultured in agarose 3D Petri dishes (100). Amirpour et al. employed a defined neural induction medium with small molecules to directly differentiate hASCs into anterior neuroectodermal cells using hanging drop protocols (132). Additionally, Salehi et al. conducted a comparison between two differentiation protocols for the generation of retinal precursor-like cells in vitro: hASCs monolayer culture and hanging drop culture with a defined medium. The study indicated that the hanging drop method led to an enhanced yield of retinal precursor differentiation, resulting in precursor-like cells that exhibited responsiveness to the glutamate neurotransmitter (133). Moreover, the hanging drop method was found to enhance the efficiency of hASC smooth muscle differentiation and improve cell viability within a 3D bioprinted structure (104). Bagheri-Hosseinabadi et al. observed a higher rate of cardiomyogenic differentiation in hASCs cultured in a 3D hanging drop system with 5-azacytidine compared to the 2D culture (134).

These findings suggest that the 3D environment may offer enhanced stimuli for the differentiation of ASCs into various lineages. These results have implications for the development of protocols for preparing ASCs for use in clinical studies focused on regeneration.

#### 4.2.3 Paracrine secretion

The paracrine secretion of cytokines such as angiogenic factors, adipokines, neurotrophic factors, and interleukin plays a crucial role in the therapeutic application of ASCs by promoting tissue regeneration and repair (120).

3D cultured ASCs possess distinct and inherent characteristics independent of the method of formation. The size of 3D cultured ASCs is a critical factor, as larger cells exhibit higher levels of hypoxic factors that stimulate angiogenesis and antiapoptotic gene expression (26). small spheroids of average spherical shape were generated in 96-well plates. The 3D condition of the hASCs was found to be correlated with elevated levels of VEGF-A and IL-8 expressions in relation to wound healing (135). Kim et al. introduced HES as a comprehensive platform capable of facilitating the rapid formation and cultivation of a substantial quantity of size-adjustable 3D hASC spheroids. Notably, HES-derived spheroids exhibited a higher VEGF secretion compared to spheroids cultured on a commercially available low-attachment culture plate. Utilizing these advantages, HES-based spheroids were employed for 3D bioprinting, resulting in enhanced retention and VEGF secretion within the 3D-printed construct compared to a similar structure containing single cell suspension (116). Yu et al. utilized agarose microwells to seed hASCs, generating uniform cell spheroids with adjustable size, and stimulated ECM deposition through the use of ascorbic acid 2-phosphate to form ASC sheets. Transcriptome sequencing analysis indicated upregulation of angiogenesis-related genes in ASC spheroids compared to monolayer ASCs. The study illustrated the stimulatory impact of spheroid formation on ASCs towards endothelial lineage by observing increased expression of cluster of differentiation (CD) 31, which persisted following the seeding of ASC spheroids on cell sheets. Furthermore, compared to ASC sheets, ASC spheroid sheets exhibited heightened expression of VEGF and HGF, and the conditioned medium from ASC spheroid sheets significantly promoted tube formation of endothelial cells in vitro (136).

Seo et al. innovatively created a switchable water-adhesive, super-hydrophobic nanowire surface to enhance cell-cell and cell-matrix interaction, leading to improved cell viability and paracrine secretion of VEGF in hASC spheroids. The size of hASC spheroids can be easily manipulated on this surface. Accordingly, the spheroids generated on this surface demonstrate significantly heightened angiogenic effectiveness in comparison to spheroids produced through traditional methods such as spinner flask suspension culture and hanging drop culture on a petri dish (112). The successful establishment of a 3D co-culture model utilizing HA gel and a 10:1 ratio of late-passage hASCs and endothelial colony-forming cells resulted in increased secretion of cytokines, including HGF, VEGF, and epidermal growth factor (EGF), compared to single-cell 3D culture or monolayer culture (109). These findings suggest potential applications of 3D strategies in angiogenesis and regeneration therapies.

Furthermore, Zhang et al. utilized a low-adhesion cell culture plate to generate rat ASCs (rASCs) into microtissues in vitro. They employed grafts composed of microtissues and polycaprolactone nerve conduit for the purpose of repairing sciatic nerve defects in rats. Their study revealed that microtissues promote the secretion of nerve regeneration-related cytokines, including brain-derived neurotrophic factor, and nerve growth factor, the angiogenic factor such as VEGF, as well as anti-inflammatory cytokines such as IL-4, IL-10, and IL-13. This secretion ultimately facilitated the growth of axons when compared to an equivalent number of cells cultured in a 2D manner (137). Zhou et al. utilized a hanging drop method to generated murine ASCs-based microtissues, which were subsequently injected into streptozotocin (STZ)-induced diabetic rats for the treatment of erectile dysfunction. The findings demonstrated elevated expression of VEGF, nerve growth factor, and TNF-stimulated gene-6 within the microtissues, indicating neuroprotective and anti-inflammatory properties (138).

Overall, the use of specific culture media and 3D cultured techniques can enhance the differentiation potential and paracrine secretion of ASCs. Hence, it is imperative to carefully deliberate on the selection and refinement of techniques for producing 3D cultured ASCs, as they have the potential to impact the characteristics of cells.

## 5 The potential of 3D cultured ASCs for diabetic therapy

Diabetes as a multi-organ disease, is a significant cause of increased morbidity and mortality worldwide. In the treatment of diabetes and its complications, ASCs have been used due to their inherent attributes such as self-renewal capacity, differentiation potential, homing mechanism and immunosuppressive property (11, 21, 139). Currently, the clinical trials of ASCs for treating diabetes and its associated complications, including the diabetic foot ulcer (DFU), diabetic critical limb ischemia, and diabetic nephropathies, are still in the preliminary research stage (**Table 2**). There is a lack of agreement regarding the optimal method of administration to achieve enhanced therapeutic outcomes. Potential routes of administration include intravascular injection, local tissue injection, and thymus injection. In diabetic patients, the most commonly used administration routes are intraportal injection and intravenous infusion (140, 141, 143, 144). For diabetic angiopathy conditions like DFU, common delivery methods include local injection of ASCs and direct application of 3D ASC grafts onto the wound site (145–147).

**Table 2:** Completed and ongoing clinical trials of ASCs in diabetes and its complications.

Diseases	Study phases	Cell types	Heterologous/Autologous/Allogeneic	Delivery modes	Key findings/NCT number
T1DM (140)	Unknown	hASCs (supplemented with cultured bone marrow)	Allogeneic	Intraportal injection	Serum C-peptide ↑; exogenous insulin requirement ↓; Hb1Ac ↓
T1DM (141)	Unknown	hASCs and BM-HSCs	Autologous	Intraportal injection	Serum C-peptide ↑; exogenous insulin requirement ↓; Hb1Ac ↓; glutamic acid decarboxylase antibodies ↓
T1DM (142)	Unknown	hASCs and BM-HSCs	Autologous/Allogeneic	Infused into the portal, thymic circulation and subcutaneous tissue	Autologous co-infusion offered better long-term control of hyperglycemia as compared with allogenic co-infusion
T1DM (143, 144)	II	hASCs (supplemented with cholecalciferol)	Allogeneic	Intravenous infusion	The therapy without immunosuppression was safe and might have a role in the preservation of β-cells in recent-onset T1DM
T1DM	I	hASCs	Allogeneic	Intravenous infusion	NCT05308836
T1DM	I	hASCs	Allogeneic	Intravenous injection	NCT02940418
T1DM	I/II	hASCs	Autologous	Intravenous infusion	NCT00703599
T2DM	Unknown	hASCs	Autologous	Intravenous injection	NCT01453751
T2DM	I/II	hASCs	Autologous	Intravenous infusion	NCT00703612
Diabetic wound (145)	Unknown	SVF (seeded in fibrin-collagen hydrogel)	Autologous	Left on the wound	Engineered skin graft with stromal vascular fraction cells encapsulated in fibrin-collagen hydrogel were safe and accelerated the wound healing process
DFU (146)	II	hASCs (seeded in the hydrogel matrix)	Allogeneic	Left on the wound	Wound closure was achieved in the treatment group
DFU (147)	I	SVF	Autologous	Injected in the ulcer bed and periphery and along the pedal arteries	SVF could be safely used to treat chronic DFU by vascular repair and/or angiogenesis
DFU	I	hASCs (seeded in Chitosan Scaffold)	Unknown	Unknown	NCT03259217
DFU	I/II/III	hASCs (seeded in hydrogel sheets)	Allogeneic	Unknown	NCT03183726, NCT03754465, NCT04497805, NCT03370874, NCT04569409
DFU	I/II	hASCs (suspended in the fibrin solution)	Allogeneic	Left on the wound	NCT03865394
DFU	I	hASCs (seeded in the fibrin sealant)	Autologous	Left on the wound	NCT02375802
DFU	I	hASCs	Allogeneic	Injected into the subcutaneous dermo-epidermal junction and homogenously around the wound	NCT05595681
DFU	I	hASCs	Unknown	Local injection	NCT02831075
DFU	I	hASCs or SVF (both supplemented with platelet rich plasma)	Autologous	Injected intradermally at the border zone and inside of wound surface bed	NCT05610865
DFU	II	hASCs	Autologous	Injected into the periphery	NCT02092870
DFU	I	hASCs	Autologous	intramuscular injection	NCT03916211
Diabetic CLI (148)	II	hASCs	Allogeneic	Intramuscular injection	NCT04466007
Diabetic CLI	I/II	hASCs	Autologous	intramuscular injection	NCT02864654
Diabetic CLI	I/II	hASCs	Autologous	Intra-arterial infusion	NCT01257776
Diabetic Nephropathies	I	hASCs	Allogeneic	intravenous infusion	NCT04869761
Diabetic Nephropathies	I	hASCs	Autologous	Intra-arterial infusion	NCT03840343

NCT, National Clinical Trial; T1DM, type 1 diabetes mellitus; T2DM, type 2 diabetes mellitus; DFU, diabetic foot ulcer; CLI, critical limb ischemia; hASCs, human adipose-derived stromal/stem cells; Hb1Ac, hemoglobin A1c; BM-HSCs, bone marrow-derived hematopoietic stem cells; SVF, stromal vascular fraction.

“↑” represents up-regulation, “↓” represents down-regulation.

Unfortunately, their efficacy is primarily impeded by the limited expansion and survival of transplanted stem cells and their inability for proper functional integration in response to the physical environment (21, 149). Moreover, only a fraction of MSCs successfully home to the pancreas and express insulin (150). 3D culture technology provides an opportunity to fill this knowledge gap. While there is a scarcity of research on the clinical application of 3D cultured ASCs, findings from animal and cellular studies suggest the potential benefits and advantages of 3D cultured ASCs in the treatment of diabetes (**Table 3**).

**Table 3:** The characteristics of 3D cultured ASCs in diabetes and diabetic complications compared to monolayer cells.

Cell viability	Stemness	Differentiation ability	Secretion function	Constructing 3D organ models	Clinical applicability
**↑**	↑	IPCs ↑; Endothelial cells ↑	Anti-apoptotic growth factors ↑; Angiogenic and wound healing-related factors ↑; Extracellular matrix proteins ↑	such as the complex adipose tissue, pancreas, blood vessels, skin, bones, cardiac and skeletal muscles, and nerves	Having uncertainty and requiring long-term evaluation (the possible presence of undefined components; the possibility of tumor development)

3D, three-dimensional; ASC, adipose-derived stromal/stem cells.

“↑” represents up-regulation.

### 5.1 Promotion of insulin production

Type 2 diabetes mellitus (T2DM) is the most common type of diabetes, characterized by two interrelated metabolic defects: insulin resistance and pancreatic islet β-cell dysfunction. The development of T2DM is influenced by a complex interplay of genetic, environmental, emotional, and behavioral factors (151). Individuals with T2DM typically exhibit insulin resistance and gradual β-cell deterioration, resulting in insufficient insulin secretion, and consequent hyperglycemia and elevated free fatty acid levels. The resulting glucotoxicity and lipotoxicity exacerbate the dysfunction of β-cells to secrete insulin in response to hyperglycemia or oral hypoglycemic agents (16).

Type 1 diabetes mellitus (T1DM) is a chronic disease characterized by insulin deficiency resulting from autoimmune destruction of pancreatic islet β-cells, ultimately leading to hyperglycemia. Although the mechanism of T1DM in still not completely understood, it is believed to involve abnormalities in multiple immune cells, including T cells, B cells, regulatory T cells, monocytes and macrophages, and dendritic cells (152).

Given their similar outcome of pancreatic islet β-cell dysfunctions, the cell therapy as a potential strategy has attracted increased research attention. However, the transplantation of functional β-cells as a therapeutic strategy is impeded by the significant challenge of generating an adequate quantity of β-cells ex vivo and subsequently maintaining their viability post-transplantation. β-cells are susceptible to hypoxia and are prone to rapid apoptosis or damage as a result of the host immune response (153). Notably, the use of 3D cultured ASCs significantly promotes the construction and transplantation of islets and promote the insulin production.

Firstly, 3D cultured ASCs are capable to differentiate into insulin-producing cells (IPCs) to promote insulin production. For example, Khorsandi et al. found that the collagen/HA scaffold could enhance the differentiation of IPCs from rASCs. Compared to the 2D culture, the insulin release from 3D ASCs-derived IPCs showed up-regulation when exposed to a high glucose medium. The percentage of insulin-positive cells in 3D culture showed an approximately 4-fold increase compared to the 2D cultured cells (154). Ikemoto et al. developed a human recombinant peptide petaloid μ-piece 3D culture method to generate IPCs from hASCs. Following transplantation of 96 IPCs under the kidney capsule or intra-mesentery in STZ-induced diabetic nude mice, the hyperglycemic state was restored to normoglycemia (155). Ohta et al. found that blood glucose levels of STZ-induced diabetic nude mice were normalized after transplantation of 3D-cultured IPCs (156).

Secondly, the immunomodulatory action of 3D cultured ASCs can improve the micro-environment of islets. Abadpour et al. developed 3D-printed bioactive scaffolds containing islets and hASCs by combining alginate and nano-fibrillated cellulose bioink. Bioink diffusion properties were demonstrated, as well as benefits of hASCs for glucose sensing, insulin secretion, islet viability, and the reduction of pro-inflammatory cytokines, including growth-regulated protein-α and interferon gamma-induced protein-10 (157).

Furthermore, the 3D culture methods can also facilitate the survival of pancreatic islets and increase the functionality of grafts before transplantation. Jun et al. introduced a method of transplantation by co-culturing single primary islet cells with rASCs in concave microwells. These spheroids exhibited distinct ultrastructural morphologies, increased viability, and enhanced insulin secretion compared to mono-cultured islet spheroids, suggesting that ASCs may protect islet cells from damage by releasing anti-apoptotic growth factors. Additionally, the co-encapsulation of islets with additional ASCs within microfibers could further prolong graft survival through the anti-inflammatory properties of ASCs (22). Wang et al. effectively produced viable and functional heterocellular islet micro-tissues by combining islet cells, human umbilical vein endothelial cells, and hASCs within porcine decellularized ECM. These 3D islet micro-tissues exhibited sustained viability and normal secretory function, as well as heightened drug sensitivity during testing. Additionally, the utilization of 3D islet micro-tissues resulted in improved survival rates and enhanced graft function in murine models of diabetes (158).

In conclusion, 3D cultured ASC grafts play more significant role of insulin production through differentiating into IPCs, improving the micro-environment of islets, and enhancing survival and functionality.

### 5.2 Treatment of diabetic foot ulcer

Diabetic complications are mainly caused by high-glucose-induced cellular and molecular impairments and dysfunctions of cardiovascular and neural systems. While monolayer ASCs have demonstrated efficacy in treating a range of diabetic complications (139), the current studies about treatment of 3D ASCs are mainly focused on the DFU.

The diabetic foot ulcer, considered among the most severe types of diabetic wounds, has significant challenges to healing due to diabetic neuropathy, reduced blood flow, and infections (159). Non-healing ulcers may progress to gangrene, requiring foot amputations.

The normal wound healing process is characterized by four stages: hemostasis, inflammation, proliferation, and remodeling. In the hemostasis stage, vasoconstriction, platelet aggregation, and recruitment of circulating coagulation factors occur. The inflammation stage involves the gathering of inflammatory cells that secrete inflammatory factors like matrix metalloproteinase (MMP) and neutrophil extracellular traps (NETs). During the proliferation stage, the inflammation diminishes, and skin cells such as keratinocytes secrete EGF, proliferate, and migrate to the wound bed. During the process of tissue remodeling, new tissue is restructured and deposited via ECM and neovascularization, facilitated by fibroblasts secreting FGF and vascular endothelial cells secreting VEGF (160–162).

In diabetic wounds, tissue ischemia, hypoxia, and a high glucose microenvironment disrupt the normal progression of these healing stages, leading to delayed or non-healing of wounds and various clinical complications (163). Currently, DFUs are treated with vascular intervention therapy, drugs and other non-surgical therapies, such as dressing adjuvant therapy, hyperbaric oxygen therapy, hyperthermia and growth factor therapy (164). However, the efficacy of these approaches remains limited (159). Therefore, future research endeavors are anticipated to concentrate on more effective treatment strategies, with a particular emphasis on advancing stem cell-based therapies.

ASCs exhibit significant promise in the treatment of diabetic foot ulcers. Basically, the effects of ASCs rely on their promotion of immunomodulation, neovascularization and fibro synthesis (165, 166). The routes of delivery of ASCs into the wound vary between direct injection (such as intradermal injection around the wound, intra-fascial, and intramuscular injection), topical gel treatment, engineered skin graft sheet, and with scaffolds. The survival rate and potency of expansion of ASCs in wound bed are limited in traditional injection. Therefore, scaffolds cell delivery systems are necessary which offer optimal environments for cell adhesion, proliferation, and differentiation (167, 168).

A common solution involves seeding cells into hydrogels. Zeng et al. proposed that gelatin microcryogels (GMs) presented a novel method of cell delivery that could not only enhance wound bed healing but also directly influence the basal layer of the wound. They demonstrated that GMs provided an enhanced microenvironment for inducing endothelial cell differentiation of hASCs, thereby offering potential in vivo applications for angiogenic regeneration. Additionally, they demonstrated the priming effects of GMs on the upregulation of stemness genes and improved secretion of crucial growth factors in hASCs for wound healing, such as VEGF, HGF, basic fibroblast growth factor (bFGF), and platelet-derived growth factor BB (PDGFbb) (169). Feng et al. examined the therapeutic potential of hASCs cultured as micro-spheroids in the HA gel. Diabetic ulcers in mice with hASC spheroids resulted in accelerated wound epithelialization and increased dermal thickness, surpassing the outcomes observed with vehicle alone or monolayer-cultured ASCs (170). An injectable hydrogel system based on PEG and gelatin was examined for delivering hASCs into diabetic wounds. The stemness-linked transcription factor expression of hASCs was preserved in vitro and cell retention was significantly enhanced in vivo by this gel. In diabetic mice, this ASC-hydrogel treatment reduced inflammatory cell infiltration, enhanced neovascularization, and sped up wound closure (23).

There are also other bioengineering approaches for constructing 3D cultured ASCs. For example, Tyeb et al. introduced a combinatorial method involving the utilization of gelatin-sericin (GS) scaffolds coated with laminin (GSL). GS scaffolds provided enhanced protection against free radical-induced damage compared to gelatin scaffolds and consequently improved cell viability and metabolic function. The utilization of rASCs loaded onto GSL scaffolds resulted in enhanced regeneration, collagen remodeling, and increased expression of CD31 in diabetic ulcer rat models (171).

However, the broad use of matrix components aiding in the formation of 3D structures may impose constraints on the clinical applicability owing to the presence of undefined components. The implementation of hASCs formulated as multicellular aggregates without scaffolds also facilitated the healing wounds of diabetic mice. These aggregates exhibited a noteworthy increase in the production of extracellular matrix proteins including tenascin C, collagen VI α3, and fibronectin, as well as the secretion of soluble factors including HGF, MMP-2, and MMP-14 when compared to monolayer culture (172).

Considering that the main mechanism of cell action involves the paracrine effect, the characterization of components secreted by cells is vital, which indicates that ASCs can also function through their conditioned media. Lee et al. successfully fabricated an alginate-based scaffold using 3D printing and electrospinning techniques, which served as a structure to encapsulate hASC spheroids. This structure not only securely entrapped the spheroids but also facilitated the stable release of factors associated with angiogenesis and wound healing, such as CD31, VEGF, HGF, C-X-C chemokine receptor type 5 (CXCR5), IL-8, and MMP-1. They also demonstrated the role of these factors through a tube-forming assay and found that conditioned media from the spheroid-scaffold group enhanced the formation of capillary-like structures in human umbilical vein endothelial cells when compared to the single cell-scaffold group (173).

Utilizing diverse 3D culture techniques and materials such as hydrogels, bioactive scaffolds, scaffold-free methods, and conditioned media from 3D cultured cells, ASCs have the potential to facilitate diabetic wound healing by the promotion of immunomodulation, neovascularization and fibro synthesis.

### 5.3 Modelling tissues and organs

Aside from their application in diabetic therapy through transplantation, 3D cultured ASCs are crucial in the development of in vitro models that mimic the pathophysiology of different tissues and organs linked to diabetes and its associated complications. These models also potentially serve as valuable tools for screening novel therapeutic interventions and minimizing the reliance on animal experimentation.

Adipose tissue is a significant location of insulin resistance in individuals with type 2 diabetes mellitus (T2DM) and is linked to heightened chronic inflammation. The establishment of in vitro models for investigating the pathogenesis of adipose tissue in metabolic diseases would offer significant benefits. Numerous efforts have been made to create 3D adipose cultures utilizing ASCs. For instance, hASCs were cultivated on plates coated with ELP–PEI copolymer, as the PEI component promotes spheroid formation and the ELP component facilitates the attachment of spheroids to the surface. This culture platform enabled the production of functional adipocytes that exhibited a favorable response to fatty acid stimulation (126). Moreover, Gerlach et al. utilized multicompartment hollow fiber-based bioreactor technology to generate 3D adipose tissue. In vitro, 3D bioreactors allowed greater metabolic activity compared with traditional 2D cultured hASCs and enabled the generation of adipose tissue as long as two months (174). Yang et al. created a 3D human adipose microtissue engineered within a microfluidic system (175). Furthermore, culture technologies have been employed in the generation of beige or brown adipose tissue (176–178). As the characterization of ASCs can be influenced by the source of adipose tissue, the availability of such tools presents a wide range of opportunities in vitro studies. By utilizing these models, it becomes feasible to compare relative metabolic responses of adipose depots under different health conditions to metabolic researches.

An ideal and comprehensive adipose tissue models should include all in vivo components, such as adipocytes, connective tissues, veins and nerves. For example, Lau et al. described an adipose micro-physiological system that involved sandwiching human WAT between tissue-engineered sheets of ASCs. The use of ASCs provided a structural ECM framework to encompass and support the mature adipocytes as well as paracrine growth factors (179). One common approach in the generation of vascularized adipose tissue involves the inclusion of exogenous endothelial cells through co-culture (180–182). The utilization of vascularized adipose models presents a promising avenue for developing novel drugs to treat metabolic diseases by modulation of the adipose vasculature. Moreover, adipose depots could be infiltrated with inflammatory and immune cells during preparation or after differentiation into adipocytes (183), offering a valuable tool for immune–metabolic research.

Furthermore, through differentiation and secretory capabilities of ASCs, it becomes possible to connect them with micro-physiological systems representing other organs. Despite being in the early stages of development, 3D models simulating organs such as the pancreas, blood vessels, skin, bones, cardiac and skeletal muscles, and nerves (**Table 4**), exhibit promising potential in mimicking the effects of diabetes and its complications, as well as evaluating the efficacy of cell transplantation therapy.

**Table 4:** Examples about 3D cultured ASC models of various organs except adipose tissues.

Model	Methods or Materials	Mechanisms	Potential application
Pancreas (22, 157, 158)	Co-culturing single primary islet cells with ASCs in concave microwells; 3D decellularized ECM hydrogels; 3D-printed bioactive scaffolds	Improve islet cell survival and function; improving local ischemia and inadequate angiogenesis; improving the islet micro-environment	Developing bioartificial pancreas; islet transplantation for diabetic therapy
Blood vessel (184–186)	Seeding ASCs in hydrogels with growth factors; seeding ASCs in agarose microwells to generate cell spheroids; bioactive scaffolds	Releasing angiogenic-related factors	Angiogenesis and regeneration therapies; ischemic muscle repair
Skin (187, 188)	Seeding ASCs in collagen sponge scaffolds; 3D decellularized ECM hydrogels	Better differentiation to keratinocytes; promoting faster neovascularization, collagen secretion, and remodeling; better cell delivery	Full-thickness skin regeneration; wound healing
Bone and cartilage (189–198)	3D cultured ASC spheroids; Embedding the pre-differentiated cells in a 3D collagen matrix; 3D-printing	Recreating the physiological bone microenvironment and promoting differentiation into osteoblastic and chondrogenic cell linages	Personalized bone tissue engineering; treating bone defects; human-engineered auricular reconstruction; regenerative therapies of cartilage defects
Muscle (199, 200)	Inducing 3D cellular alignment and using dynamic biomimetic culture of ASCs; seeding ASCs in chitosan membranes	Enhancing myogenic differentiation capacity	Developing 3D skeletal muscle grafts; treating myocardial infarction
Nerve (201)	Seeding ASCs in bioactive hydrogel scaffolds	Enhancing differentiation into neurons	Developing neural tissue engineering
Liver (202)	Seeding cells in microporous hydrogel scaffolds with hexagonally packed interconnected cavities and ECM	Enhancing hepatic differentiation	Hepatocyte transplantation; establishing human physiologically relevant liver models in vitro

3D, three-dimensional; ASC, adipose-derived stromal/stem cells; ECM, extracellular matrix.

## 6 Conclusions and future prospects

3D cultured cells have been advantageous in various biomedical fields. This technology is still in the early stages. The isolation, culture, and identification of cells are the basis of 3D culture. Therefore, large-scale manufacturing methods incorporating quality control are necessary for producing cells and 3D cultured transplantations.

Indeed, 2D adherent cell culture of ASCs is still conventionally used for both in vitro and in vivo studies. These cells have been extensively characterized, whereas many factors have not been analyzed on 3D cultured ASCs yet. Additionally, while monolayer ASC cells have shown effects for the treatment of diabetes and its complications in both clinical trials and animal experiments, current research status on 3D cultured ASCs mainly concentrates on T1DM and DFU only. Thus, further research is required to better understand the function and underlying mechanisms of 3D cultured ASC therapy.

The potential side effects of ASCs for tumor development should not be disregarded in studies; however, they may also serve as a potential tool for antitumor therapies. While tumor cells altering the phenotype and function of in vitro cultured ASCs through paracrine mechanisms (203), ASCs can also serve as a factor that promotes tumor growth (203–205). By contrast, ASC exosomes were shown to possess immunomodulatory properties and can inhibit cancer growth, migration, and colony formation (206). A strategy for tumor therapy used ASCs which loaded gold nanorod (AuNR)-PEG-poly(ethyleneimine) (APP) and Chlorin e6 (Ce6). Following activation of the APP/Ce6 agents through irradiation, ASCs were shown to play a role in tumor migration, tropism, and exhibit anticancer properties (207). These findings underscore the importance of exercising caution in the utilization of 3D cultured ASCs, with long-term experiments necessary to assess their safety. Specifically, careful consideration should be given to the potential of 3D culture to induce tumorigenesis while enhancing cell viability and stemness.

## Author contributions

YS: Conceptualization, Writing – original draft. XY: Conceptualization, Writing – original draft. JM: Writing – review & editing. WK: Writing – review & editing. XH: Writing – review & editing. JZ: Writing – review & editing. LC: Project administration, Supervision, Writing – review & editing.
